# The use of FDG-PET in diffuse large B cell lymphoma (DLBCL): predicting outcome following first line therapy

**DOI:** 10.1186/s40644-014-0034-9

**Published:** 2014-11-29

**Authors:** Monica Coughlan, Rebecca Elstrom

**Affiliations:** Genentech, Inc., 1 DNA Way, South San Francisco, CA 94080 USA; Stanford University School of Medicine, Palo Alto, CA 94305 USA

**Keywords:** Lymphoma, Surrogate endpoint, Positron emission tomography

## Abstract

Positron emission tomography (PET) using ^18^fluoro-2-deoxyglucose (FDG) has become a standard clinical tool for staging and response assessment in aggressive lymphomas. The use of PET scans in clinical trials is still under exploration, however. In this review, we examine current data regarding PET in DLBCL, and its potential applicability to development of a surrogate endpoint to expedite clinical trial conduct. Interim PET scanning in DLBCL shows mixed results, with qualitative assessment variably associated with outcome. Addition of quantitative assessment might improve predictive power of interim scans. Data from multiple retrospective studies support that PET-defined response at end of treatment correlates with outcome in DLBCL. Optimal technical criteria for standardization of acquisition and criteria for interpretation of scans require further study. Prospective studies to define the correlation of PET-defined response and time-dependent outcomes such as progression free survival (PFS) and overall survival (OS), critical for development of PET as a surrogate endpoint for clinical trials, are ongoing. In conclusion, evolving data regarding utility of PET in predictcing outcome of patients with DLBCL show promise to support the use of PET as a surrogate endpoint in clinical trials of DLBCL in the future.

## Introduction

The noninvasive, three-dimensional, functional imaging technique known as positron emission tomography (PET) has become an increasingly powerful tool in clinical oncology. Throughout the past decade, a wealth of data have been gathered regarding the role of PET in lymphoma, and PET has taken a prominent role in clinical care of patients with lymphoma. An ongoing challenge is to put these data to use in clinical trials, whether as a surrogate endpoint or as an aid in early drug development. PET images show snapshots of biochemical processes *in vivo* by relying on injected compounds labeled with positron emitting radionuclei [1]. ^18^Fluoro-2-deoxyglucose (^18^F-FDG or FDG), a glucose analog, is the most commonly used PET tracer in oncology, and references to PET throughout this article refer to FDG-PET. After injection, FDG is taken up by cells and subsequently phosphorylated in the same process as glucose. The phosphorylated product, however, is not further metabolized and remains trapped in the cells for extended periods of time [2]. This accumulation of FDG is detected by PET, and provides visualization of glucose consumption by different cells. The fact that metabolism in cancer cells is fundamentally altered was first described by Warburg in the 1920s, and the mechanisms by which this deranged metabolism occurs has been the subject of extensive investigation, which has illuminated a variety of oncogenic pathways that contribute to the effect [3].

This fundamental requirement for cancerous cells to upregulate their glucose metabolism allows PET to be a useful tool in oncology. Cancerous cells show increased amounts of the membrane glucose transporters, GLUT-1 and GLUT-3, increased hexokinase, and overall augmented glucose metabolism [3,4], making FDG an optimal tracer for measuring viability of malignant cells. When combined with the anatomical imaging tool computed tomography (CT), PET/CT scans give more accurate images that can discriminate between physiological and pathological FDG uptake, as well as between necrotic masses and viable tumors. The increased accuracy of combined PET/CT scans underlies the utility of PET for the staging, monitoring, and restaging of many cancers including non-small cell lung, colorectal, esophageal, and head and neck cancers, melanoma, as well as lymphoma [5]. The role of PET scanning using FDG in lymphoma, particularly regarding the use of PET in optimizing conduct and interpretation of clinical trials for DLBCL, will be the focus of the remainder of this review.

## Review

### Biological and technical parameters- what influences the final scan?

Both biological and technical factors can influence the final PET image (Table 1). Many of the biological factors can vary from patient to patient, making them difficult to control. These biological factors, such as amount of and location of brown fat, post-chemotherapy thymic rebound, the use of granulocyte macrophage-colony stimulating factor, inflammation, and infection, can have a confounding effect on PET images. These factors are often falsely identified as active disease on PET images because they cause FDG uptake in regions not compatible with normal physiological FDG uptake [6]. A patient with lymphoma, for example, who has brown fat around the cervical lymph node region, could be incorrectly identified as having active disease because the brown fat also has a high level of FDG uptake.

**Table 1:** **Biological and technical factors affecting PET images**

**Biological:**	**Technical:**
Any process associated with increased glycolysis	Scan acquisition parameters
	• scan duration
•Brown Fat	• bed overlap
•Inflammatory cause	• FDG dose
•Hyperplasia in the bone marrow post granulocyte colony-stimulating factor	Image reconstruction and spatial resolution of scanner
•Patients motion/breathing	Timing of PET scan after FDG administration
•Patient comfort	ROI strategy and normalization calculation
•Blood glucose level	Use of contrast agents during CT

Technical components of the scanning technique that influence the final image include factors such as FDG dose, image reconstruction, and spatial resolution of the scanner [7]. The procedures guiding these components can vary across scanners and institutes. With the growing use of PET in lymphoma and other cancers, and as a prerequisite to establishing PET as a possible surrogate endpoint in clinical trials, there is a need to standardize patient preparation and scan acquisition, as variability in these areas can impact the quality and reproducibility of scan interpretation across centers. This variability could impact the results within a multi-center study, as well as make the comparison of results across studies difficult. Several standardized protocols have been developed for use in clinical trials. In an attempt to standardize whole-body PET for multi-center clinical trials in the Netherlands for example, the Dutch Society of Nuclear Medicine implemented a protocol that included patient preparation, administration procedure, FDG dosing, resolution matching, data analysis, standardized uptake value (SUV) normalization, and quality control approaches [7]. This protocol was designed in an attempt to minimize errors in obtaining SUV measurements so that these SUV results could be interchangeable across centers [8].

A UK-based group published another example of a standardized PET protocol with the goal of ensuring the acquisition of quality scans in UK trials. This group established a clinical trials network (CTN) that PET centers could join by adhering to quality control and the specified acquisition procedure. A “core laboratory” was set up for quality control procedures and scan interpretation in order to obtain uniformity and high-quality results across the CTN. The CTN has been used in three multicenter trials examining the utility of PET in Hodgkin lymphoma (HL), and thus far has been shown to be an effective method of ensuring compliance across PET centers [9].

A Japan-based multicenter trial evaluating the utility of interim PET scanning in DLBCL patients included optimization of the scan acquisition protocol and image reconstruction parameters. The study group implemented quality control measures in order to determine the optimal parameters for each PET scanner in the trial. They also established a trial-specific core laboratory to evaluate the effectiveness of these measures across the centers, and the standardization method was shown to be successful in obtaining quality images [10].

The protocol standardization exemplified by the Dutch, UK, and Japanese study groups is a model that other multi-center studies can follow. In the United States, there have also been published protocols to standardize patient preparation and scan acquisition. The Cancer Imaging Program of the National Cancer Institute developed a PET standardization protocol in 2006 for use in NCI trials. The NCI recommendations cover patient preparation, methods of scan interpretation, quality assurance, image acquisition and reconstruction guidelines, as well as other technical parameters [11]. These recommendations offered a starting point for the standardization of PET methodology for the use of FDG-PET as a biomarker for treatment response in clinical trials [12]. Around the same time, detailed guidelines were published with the purpose of assisting physicians in utilizing combined PET/CT for oncologic imaging [13]. One challenge to scan standardization in countries where PET scans are more widely available due to the use of mobile PET scanners, such as in the US, is that the variability across this type of scanner and the impact of their constantly changing environments increase the challenges in maintaining consistent quality control. Despite this difficulty, standardization of patient preparation, scan acquisition, and quality control are becoming components of many current PET studies, improving quality and contributing to more reproducible PET results.

### PET in lymphoma- when do we use it?

Oncologists increasingly use PET in the routine staging of many types of lymphoma to clarify the extent of disease involvement and to help direct treatment options [14]. With few exceptions, such as small lymphocytic lymphoma (SLL), the vast majority of malignant lymphomas, including both aggressive and indolent subtypes, are reliably FDG-avid, and therefore detectable on PET scanning [15–17]. Although a thorough discussion of lymphoma staging using PET is outside the scope of this review, detection of bone marrow involvement merits mention, given its relevance to clinical trial procedures and assessments. The probability of involvement of bone marrow in lymphoma varies by histology, and can have an impact on both prognosis and management. Assessment of lymphoma involvement of bone marrow is historically done by iliac crest aspirate and biopsy, a procedure that is often uncomfortable for patients, and provides only local assessment of potential involvement. While the utility of CT scanning is limited to detection of destructive bone lesions, PET is able to detect involvement of bone or bone marrow in many cases [16–18]. Several studies have addressed the question of whether PET and bone marrow biopsy both contribute relevant staging information. It is clear that PET scanning detects focal lesions that cannot be identified by iliac crest biopsy. However, the added information provided by bone marrow biopsy in the setting of PET scan has been a subject of controversy. This controversy could be due in part to inclusion of differing histologic subtypes of lymphoma; notably, recent studies including only DLBCL and using modern PET technology continue to show divergent results regarding additional clinical and prognostic information obtained from BMB [19–22].

Since the publication of the 2007 Revised Response Criteria for Malignant Lymphoma [23] (subsequently updated as the Lugano Classification [24]), end of treatment response assessment for FDG-avid lymphomas routinely incorporates PET results. PET has also been explored in assessment of early or interim response to treatment and to predict post-treatment prognosis [25].

Interim PET imaging refers to imaging performed during a planned course of therapy, prior to completion, with the goal of predicting the outcome of that course of treatment. If interim imaging can predict early in the course of therapy that a specific treatment will not be efficacious, therapy can potentially be altered prior to completion of the entire course. This could spare patients toxicity of non-efficacious therapy, and allow revision to a potentially more effective regimen in patients who are destined to respond poorly to the initial treatment.

Interim PET imaging has been explored most extensively in Hodgkin Lymphoma (HL), but has also been examined in DLBCL and FL. In DLBCL, although many patients are cured, those patients who do not respond to or who relapse after standard first line therapy have poor survival [26]. Therefore, if interim PET could predict response to treatment or survival, patients who are responding poorly to therapy might be identified early in the treatment course. If a patient were found to have a poor chance of achieving good results from the current treatment regimen based on interim PET, the therapy could then potentially be altered in hopes of achieving a better outcome. A caveat to this use of PET is that the interpretation of interim PET should have low rate of false positives, in order to avoid altering a patient’s treatment from a truly effective regimen.

Although end of treatment PET scan results are routinely used for response assessment in clinical practice, PET scans have not yet been systematically incorporated into clinical trial assessments. The time frame for end of treatment PET imaging is 6-8 weeks after the completion of therapy [23], and the median PFS for frontline DLBCL patients is approximately 5 years [27]. If the end of treatment PET result could be systematically correlated with PFS or overall survival, then PET could potentially be used as a surrogate endpoint in clinical trials. This would effectively shorten clinical trials, thereby making novel treatment for patients available sooner.

### PET interpretation criteria

There has been much debate about the appropriate method of interpreting PET scans at both the interim and end of treatment time point. As mentioned earlier, the Revised Response Criteria for Malignant Lymphoma incorporates PET scans performed at the end of treatment as a major determinant of response. The criteria that were developed for interpreting end of treatment PET scans for malignant lymphomas are known as the International Harmonization Project, or IHP, criteria. These criteria depend on a visual comparison of FDG uptake in the large residual lesions to that of the mediastinal blood pool (MBP), and of FDG uptake in smaller lesions, less than 2 cm in diameter, to that of the surrounding background [28] (Table 2). Because of their incorporation into the 2007 standard Response Criteria [23], this method of evaluation is commonly used in assessment of end of treatment PET scans.

**Table 2:** **Criteria for PET interpretation**

**International Harmonization Project** **(IHP)** **criteria (adapted from Juweid 2007) [** 28 **]:**	**Deauville criteria (Meignan 2009) [** 29 **]:**
*Definition of a positive scan*:	1. No uptake
Focal or diffuse FDG uptake above background in a location incompatible with normal physiology	2. Uptake ≤ mediastinum
*Exceptions*:	3. Uptake > mediastinum but ≤ liver
1.) Mild FDG uptake at the side of moderate to large residual masses with lower intensity visually compared to the mediastinal blood pool structures.	4. Uptake moderately > liver uptake, at any site
2.) FDG uptake in residual hepatic or splenic lesions should be compared to the uptake in surrounding normal liver or spleen	5. Markedly increased uptake at any site and new sites of disease.
	A positive scan is usually interpreted as one showing areas of residual uptake with a score of ≥4, though criteria may vary

A second criterion that has been used to interpret PET scans is a five-point visual scale known as the Deauville criteria (Table 2). The Deauville criteria, also known as the London criteria, were first used for interpretation of interim PET scans in HL. These criteria were shown to provide a robust and reproducible method of interpreting interim scans in HL, and are currently the reference standard in a study designed to validate the utility of interim PET in HL [9]. In 2009, at the annual international workshop for interim PET in DLBCL and HL held in Deauville, France, this five-point visual scale was proposed for use in DLBCL in addition to HL [29], and has recently been incorporated into the updated standard response criteria for lymphoma (The Lugano Classification) [24]. The Deauville criteria depend on a visual comparison of FDG uptake in regions of interest to that of the liver, which generally shows higher FDG uptake than the MBP. This difference in reference background organ represents a key differentiator between the Deauville and the IHP criteria, with the Deauville having a higher tolerance of residual uptake than the IHP.

A third approach to scan interpretation uses changes in quantitative measures of FDG uptake from baseline to interim or end of treatment scans to identify responders and non-responders to therapy. Standardized uptake value (SUV) is a semi-quantitative measurement of intensity of FDG uptake in a given area of a scan. One variable that is under investigation as a response tool is the change in maximum standardized uptake value (ΔSUV_max_). Other quantitative variables under exploration include ΔSUV_mean_, total lesion glycolysis, and metabolic tumor volume (Table 3). These quantitative approaches to interpreting scans have shown promise in minimizing reader bias and improving reproducibility; however, these quantitative analyses also require more stringent scan acquisition protocols.

**Table 3:** **Definitions of quantitative FDG measurements**

**Measure**	**Definition**	**Notes**
Standardized Uptake Value (SUV) [30]	Relative tissue FDG uptake adjusted for amount injected and body weight	Variable based on injected tracer, time of uptake
SUV_max_ [30]	Hottest pixel in defined region of interest (ROI)	
SUV_mean_ [30]	Average SUV within a ROI	May vary depending on operator definition of ROI
Metabolic Tumor Volume (MTV) [31,32]	Total volume of lesion pixels exceeding a threshold value	Dependent on definition of ROI
Total Lesion Glycolysis (TLG) [32]	Product of MTV and SUV_mean_	

### Interim PET in DLBCL

Interest in the use of interim PET in DLBCL stems in part from observations that suggest excellent prognostic power of interim PET in HL. A number of small studies in the last decade suggested that PET scan results obtained after one to 3 cycles of chemotherapy correlated well with outcome in both HL and DLBCL [33–35]. In 2007, a study evaluating interim PET results in patients with HL demonstrated that interim PET was a powerful predictor of outcome in patients with advanced HL [36]. Patients who were identified as having a positive PET scan at interim showed consistently dismal outcome to standard therapy, whereas those with negative scans showed excellent rates of cure, demonstrating high positive and negative predictive power in this patient population. The Response Adapted Treatment in Hodgkin Lymphoma (RATHL) trial is an ongoing follow up study that is designed to further establish the prognostic capabilities of interim PET in HL [9]. Both the strong data in support for using interim PET scanning in HL and the establishment of a reproducible, standardized method of interpretation in this indication have set the bar for interim PET in DLBCL.

In contrast to HL, although early data in support of the use of interim PET scanning in DLBCL were promising, more recent studies, conducted in the era of standard rituximab use in NHL, have shown poor predictive power of interim PET in DLBCL. One potential reason for this discrepancy is based on the ability of rituximab to recruit inflammatory cells to sites of disease. These inflammatory cells have a high rate of glucose metabolism and therefore are FDG-avid, resulting in false positive results on PET [37].

Several studies have examined the utility of interim PET in DLBCL in the rituximab era, with conflicting results. Although some have shown good correlation of interim PET results with long term outcome [38–41], others have shown either no correlation or relatively poor positive predictive power [41–45], limiting the clinical utility of the findings. One of the first to address this issue, by Moskowitz and colleagues, showed no difference in outcome between patients with a negative vs. a positive PET after 4 cycles of R-CHOP chemotherapy. While the generalizability of this finding was questioned based on a dose-dense chemotherapy regimen and the fact that all patients underwent a change in therapy after the PET scan, this study raised the possibility that DLBCL might be quite different from HL in terms of utility of interim PET [45].

Other studies have shown a statistical difference in outcome based on interim PET findings, but the clinical relevance of the difference has been questioned, particularly with respect to whether the positive predictive power is strong enough to prompt a change in therapeutic approach. For example, a study by Cashen and colleagues evaluating the prognostic capability of DLBCL interim PET results interpreted using the IHP criteria showed that there was indeed a statistically significant difference in EFS based on interim PET results, with 63% of patients with a positive PET free of an event at 2 years vs. 85% of those with a negative interim scan (p = 0.04). The authors, however, argued that the difference in PFS was not clinically meaningful, and that the difference in outcome between the two prognostic groups was not strong enough to alter treatment based on interim PET result [43]. Another retrospective study used the Deauville criteria to interpret interim PET scans, with the goal of determining the predictive value of interim PET for PFS in DLBCL patients. The results of this study showed only a weak correlation between interim PET result and PFS, with 85% of patients with a negative interim scan and 72% of patients with a positive interim scan progression free at 2 years (p = 0.047). This finding suggests that the two prognostic groups identified did not have a clinically meaningful difference in outcome. The rate of false positive interim PET scans, patients who were identified as PET positive at interim but then had prolonged remission, was high in this study [44].

The lack of compelling evidence for strong predictive power of positive interim PET for long term outcome has prompted some investigators to evaluate a quantitative approach, measuring changes in the semiquantitative SUV [41,46]. A retrospective analysis by Casasnovas and colleagues evaluated the optimal ΔSUV_max_ for DLBCL interim PET scans, and compared the predictive power of this cutoff to that of interpretation by modified IHP criteria [47]. The study examined the changes in maximum SUV from baseline to interim scans taken after 2 cycles of therapy (PET2) and after four cycles of therapy (PET4). At both time points, the quantitative models better predicted PFS than the visual assessments. The authors retrospectively identified a ΔSUV_max_ of >70% at PET4 as the optimal cutoff for distinguishing long term outcome and the interim PET4 results were more predictive than PET2. However, the best separation of prognostic groups was identified when combining the PET4 ΔSUV_max_ of >70% with the visual assessment . Using this strategy, the authors showed that 75% of patients with a positive PET4 and ΔSUV_max_ of ≤70% relapsed within 8 months, whereas 2 year PFS was >90% in those patients with either a negative PET4 or ΔSUV_max_ at PET4 of >70%. These results suggest that either a quantitative or a combined quantitative/visual approach might improve the predictive power of interim PET in DLBCL, although these results would require prospective validation. A caution to using the quantitative approach is that SUV tends to be more sensitive to the patient preparation and scan acquisition procedures, so relying on this variable would require more stringent standardization in protocols such as that seen in Dutch, UK, and Japanese study groups discussed earlier.

The reproducibility of results across readers and centers is another important consideration in using interim scanning. Although this standardization has been fairly effective in HL, it has been more challenging in interpretation of interim PET scans in DLBCL. A study by the Eastern Cooperative Oncology Group (ECOG) evaluating PET-based therapeutic interventions also included a substudy examining the agreement of scan interpretation across three readers when using both modified IHP criteria and the Deauville criteria [48]. In this study, agreement between readers was 68% for the modified IHP criteria (κ statistic =0.445) and 71% (κ = 0.502) for the Deauville criteria, demonstrating only a moderate level of agreement in interpretation, even between expert readers. These results are in contrast to those observed in a large Phase 3 study in HL, in which scan interpretation using the Deauville criteria was shown to be reproducible across centers (κ = 0.79 to 0.85) [9]. This finding emphasizes the need for a consistent, standardized approach to interpretation of DLBCL interim PET scans.

Overall, the utility of interim PET in DLBCL patients remains to be validated. The variety of criteria available for interpretation has made it difficult to compare results across trials, thus making it difficult to understand the role interim PET scanning might play in DLBCL. An approach incorporating quantitative measurements might be superior to a qualitative approach, but this would require a standardized scan acquisition protocol to make results reproducible, and further prospective study is required to validate this approach.

### PET at end of treatment in DLBCL

Currently, response assessment at end of treatment in clinical practice routinely incorporates PET scanning for patients with DLBCL. The 1999 International Workshop Criteria (IWC) for NHL defined responses after treatment, with a designation for complete remission/unconfirmed (CRu) to account for the difficulty in assessing the viability of residual masses with CT alone [49]. In the following years, a number of studies examined the value of PET at the conclusion of therapy in lymphoma, demonstrating that the addition of end of treatment PET results, interpreted using visual criteria, resulted in a better correlation of response with long term outcome when compared to the anatomic assessment alone [50]. A systematic review published by Zijlstra and colleagues in 2006 demonstrated that, in spite of methodological variation, the predictive power of PET at end of therapy for determining outcome in aggressive NHL was very strong [51], with reported pooled sensitivity of 72% and specificity of 100%. This prompted an effort to further standardize the use of PET in lymphoma and to integrate end of treatment PET results into lymphoma response assessment. An International Harmonization Project (IHP) developed recommendations for the interpretation of post-treatment PET scans using visual assessment, and these recommendations were incorporated into the 2007 Revised Response Criteria for Malignant Lymphoma [23]. These criteria have been the standard method of interpreting end of treatment PET scans to assess treatment response. However, these interpretation criteria were developed using data from patients treated in part before the widespread use of rituximab in the standard of care therapy for DLBCL patients.

In a study published in 2009, Han and colleagues examined the predictive power of post-treatment PET scans in aggressive B cell lymphomas treated with rituximab-containing regimens [52]. In this study, scans were assessed visually as positive or negative based on a comparison with the MBP, similar to the IHP criteria. However, the results showed poor predictive power of the end of treatment PET result, with positive predictive power of only 19%, and the two groups identified by positive vs. negative end of treatment PET result did not show significantly different PFS (p = 0.47). The authors suggested that the high rate of false positive scans was due to the use of rituximab in the patient population. Other studies performed in the rituximab era that used the IHP criteria to assess DLBCL end of treatment PET scans did show a statistically significant difference in outcome between patients identified as having a negative vs. a positive end of treatment PET scan, but the positive predictive value of these studies was variable (30-70%) [43,44]. More recently, Manohar and colleagues compared three different interpretation criteria for PET scans performed at end of therapy in patients with aggressive NHL [53]. The authors interpreted a set of end of treatment PET images using the IHP criteria, the Deauville criteria, and a semi-quantitative approach that uses an SUV_max_ cutoff of 3.5 (referred to as the Gallamini criteria) to determine which criteria best predicted outcome. The Gallamini and Deauville criteria were shown to better distinguish outcome between patients with positive vs. negative scans than the IHP, with accuracy of 88%, 84%, and 71% respectively. The authors noted that the main difference between the Gallamini and Deauville criteria and the IHP was that the IHP criteria produced a larger number of false positive results. The authors recommended the Deauville criteria over the semi-quantitative Gallamini criteria due to their simplicity in interpretation. The results from this study suggest that minimal residual FDG uptake at end of treatment is not associated with elevated chance of relapse, and that a higher threshold for determination of residual FDG uptake may be more predictive of outcome than that defined by the IHP criteria. Similar findings were reported by Martelli and colleagues, who showed that, in patients with primary mediastinal DLBCL, response assessment at end of treatment using the Deauville criteria better correlated with outcome than response assessment using IHP criteria [54].

The recommendation to use Deauville criteria at end of treatment in place of IHP criteria has been incorporated into updates of expert recommendations in PET imaging, such as that from the 4^th^ International Workshop on Positron Emission Tomography in Lymphoma [55], and into the updated standard lymphoma response criteria, the Lugano classification [24], due to its improved reproducibility and correlation with outcome in DLBCL.

### Leveraging PET in clinical trials

The clinical utility of PET scanning in lymphoma has been demonstrated in terms of both improved staging, allowing physicians to accurately plan treatment approach based on disease extent, and response assessment at end of treatment in order to optimally assess whether the patient has achieved a complete remission or needs evaluation for possible residual disease.

The impact of PET scanning on conduct of clinical trials, conversely, has been limited, as evidenced by the fact that recently-published large, randomized Phase 3 studies in DLBCL have not systematically reported or even obtained PET data [56,57]. A major reason for this lack of impact is the fact that clinical trial endpoints depend on time-dependent outcome measures, such as overall survival or its accepted surrogate, progression free survival. Response rate is not an accepted surrogate for these time-dependent outcomes, because response, particularly as defined by conventional CT criteria, is an imprecise predictor of outcome. While response as defined by PET at the end of standard first-line therapy is a better predictor of PFS than CT-defined response [50], the power of PET-defined response to predict outcome has likewise not been established. If such a predictive relationship could be established, however, the use of PET-defined response as a surrogate in studies of lymphoma could provide major advantages in terms of shortening the timelines of clinical trials, allowing results to be available faster, with commensurate earlier incorporation of these results into optimizing care for patients with DLBCL.

In qualifying a surrogate endpoint, specific criteria should be met. These include 1) that the endpoint have an accepted, standardized definition, 2) that there be data from multiple studies showing strong correlation of the endpoint with clinical outcome, 3) prospective clinical studies must validate that the surrogate is truly predictive of clinical outcome and to what extent, and 4) prospective studies should determine whether the surrogate endpoint is generalizable to other patient populations and treatments with alternative mechanisms [58]. Regarding the potential for use of PET as a surrogate in DLBCL, criterion number 2 is well supported. However, additional work is needed to define the optimal interpretation criteria (criterion number 1). Trials are currently ongoing attempting to prospectively validate PET results as a predictor of outcome (criterion 3), but the fourth point, determination of the utility of PET in the setting of treatments with differing mechanisms of action, will be an ongoing challenge. One could imagine that therapies targeting signaling pathways that directly interfere with glucose metabolism, such as PI3K inhibitors, could be less reliably associated with long term outcome, though providing an excellent measure of drug targeting.

## Conclusions

In summary, PET scanning holds great promise in both clinical treatment and facilitation of clinical trials on DLBCL. More work is needed to understand the best interpretation methods and settings for the use of PET scanning. We eagerly await studies addressing these outstanding questions.
